# Artificial Dentures

**Published:** 1863-04

**Authors:** W. H. Atkinson


ARTIFICIAL DENTURES—*By W. H. Atkinson, M. D.—Read before the Society of Dental Surgeons of the City of New York.*—The making of artificial dentures has usually been denominated "mechanical denistry," in contradistinction to "surgical dentistry," or extracting and filling teeth. To this term I object as inadequate and damaging to the standing and interests of the profession, as it presupposes that no knowledge other than abstract mechanics, or merely manual skill, is necessary to the production of the highest workmanship in the manufactures denominated artificial dentures, which is by no means the case if we include the necessary preparation of the dental arches for their reception. Each case involves the necessity of the possession of the requisite knowledge and skill to diagnose and perform the operations appropriate to this end.

Here and elsewhere we feel the need of a correct and well-digested "code of principles and practice" upon which to lean for assistance to make up correct judgment. In preparing a mouth, the majority of those who insert artificial dentures prefer a wholosale extraction of the natural teeth, especially if the shadow of disease can be conjured up pretaining to them as *carte blanche* to remove them all from their cherished lodgings; while a few at the very head of our useful profession and art advocate the restoration to a healthy and useful condition of all the natural organs remaining in the mouth, and the insertion of only as many substitutes as are necessary to complete the normal range.

The advocates of wholesale extraction are, to a man, indifferent as to the retention of the alveolar process, "especially as far as they are likely to absorb," doing more, as they say, in five minutes for the patient, in this direction, than nature can effect in six months of tedious suffering, in removing sharp spiculæ and fragments of the alveolar bodies.

Difference of opinion and practice grows out of—first, ignorance; second, inattention; third, tarrying the facts, instead of pushing on to the philosophy they indicate. Be assured, gentlemen, all are seeking for the truth, but *many* have a blundering way of coming at it. And, that I may not seem to be but a grumbler, who sees the difficulties in our pathway and lacks perception or earnestness to attain the desired goal, I will enter somewhat into the philosophy of the preparation of the arches by extraction, when this is deemed necessary, even at the risk of being possibly somewhat tedious to those who can anticipate all that *any* can say, and therefore sit in uneasiness during the circumlocution necessary to a distinct enunciation of both fact and philosophy. Extraction having been decided upon, what are the advantages of retaining the processes?

Answer. The alveolar plates ought not to be removed or fractured beyond the level of the cancelous transverse process, if possible to prevent, because they form and limit the ridge upon which to firmly fix the artificial piece, and also afford attachment to muscles and ligaments that will be materially changed if those are lost by friction or absorption.

What reason is there for removing the internal and external free borders down to this line?

Answer. Simply because it is as yet impossible to prevent absorption to this point by the most judicious and prompt methods and means of treatment.

What are the objections to awaiting nature's removal of them thus far?

Answer. First, because she does it slowly, and by a very unpleasant method; second, because it delays the insertion of a denture that can be worn with comfort, because of the manner of oxfoliation of these from their borders.

What is nature's method of removal?

Answer. Solution of the plate some distance from and not at the free edge.

Why does this necessarily occur?

Answer. Because we must have the presence of a sufficiently perfect net-work of capillaries to supply a requisite quantity of blood, affording the adequate exudate to effect the solution of the lime-salts that harden the bone, which solution frees all the ragged fragments from their connections with the body of the process. These are then dissolved by the acrid discharge from the parts which have died from the mutilation of the molecules of gum and denser parts of cartilage and bone, or become points of serious irritation, barely hanging to a slight attachment of soft parts, which usually call attention to them, resulting in their prompt removal, after which the points heal rapidly.

Is there any other objection of moment to awaiting the healing and absorption, until the arches are completely rounded off to a graceful equality of contour and level?

Answer. Yes; there is the most potent reasons against thus waiting, in the atrophy of the muscles of mastication, deglutition, and speech, all of which have to be redeveloped up to the normal standard of size and strength, *after* inserting the teeth of substitution.

If teeth of the proper size and length be inserted after absorption has been effected, without the use of a temporary set of teeth, the piece will look too full, and the lips will appear stetched and grinning for weeks or months, until the atrophied condition shall have been overcome by approximation to the natural uses of the muscles. Or, on the other hand, if we wish to avoid the inconvenience and the awkward appearances just cited, we must set smaller, shorter teeth than the conformation of the individual demands, to accommodate the shrunken jaws and lips, and thus perpetuate the expression of premature age consequent thereon. Artificial dentures are useful and ornamental in the ratio that they approximate the natural organs.

If the principles already indicated be apprehended, it will require no argument to induce each operator to carefully avoid fracture of the processes in extracting, and immediately peel off the free border of the loose gum down to a level with the transverse processes, taking an excising forceps, and cleanly and smoothly cutting away the thin margins down to the limit indicated above; then fold together the loose borders of gum, and if there be any points of excess over just enough to make a smooth, regular line of union along the summit of the alveolar ridge, trim them off to a straight line with sharp and light scissors; now fold them down till they meet, after which insert a pledget of lint, cotton, or a small napkin immediately over the line of junction, requesting the patient to close gently but firmly upon it. If there be no hæmorrhagic tendency, the pledget or other appliance may be used dry, but if requisite it may be saturated with perchloride of iron, persulphate of iron, or any of the styptics in solution, and retained a few moments, until the lips of the wound are nicely glued together; after which remove all foreign matters from the mouth and proceed to take your impression.

It is worthy of remark, and of the time it requires to describe and do this nice little operation of "peeling" the soft parts off the processes. We should secure the separation between the bone and the periosteum, always leaving the latter attached to the soft parts by the natural connections. It is not of so much moment to effect this at the very edge as it is at the point which we propose to excise the processes, for the reason, that if we fold in an already organized periosteum, calcification of the entire ridge takes place much more rapidly, and also more perfectly, than if left to the slow metamorphosis from an exudate wept out for the purpose.

Is absorption limited to this line of action?

Answer. Only in the best constitutions and best habits, for even soundness of constitutions is not proof against bad habits of life, which so potently affect nutrition. Unequal pressure, whether resultant upon imperfection of adaptation of the denture, or from persistence in the habit of biting and chewing in one locality of the dental arch, will incite absorption. Wherever the gum is thin it will be poorly nourished, and hence harden into a semi-cartillaginous state, or so encroach upon the bone as to arrest the circulation in that and cause it to absorb away, leaving but a leathery, yielding ridge, upon which the denture must now depend for retention and resistence, neither of which is so complete as where the ridge is retained in boldness of outline by a well-nourished and well-calcified bone. This condition is frequently brought about by the inequality of adaptation resulting from the use of what are called "air-chambers." Many dentists of good reputation and much skill in adapting artificial dentures make this mistake, and in consequence have to reconstruct their work, at intervals of one to five years, if they wish to maintain firmness of adaptation. So generally true is this, that it has become a prevailing doctrine among these men that the usefulness of artificial dentures does not average more than three to six years.

These results do not occur in the practice of those members of our body who do not resort to these "pets" and "pests" of dentist and patient. Probably exact equality of pressure all over the whole range upon which the piece infringes will be most satisfactory and useful for the long run, but for immediate retention of upper plates *in situ* there is no doubt that at least an approximation to a vacuum facilitates adhesion. This form of "blind chamber" (as it has been called) is a sort of compromise between excessive chambers on the one hand, and absolutely "no chamber" on the other. For the sake of peace on all sides, it may be well to use those "blind," "feather-edged," or "approximate" chambers, as they soon resolve themselves into equal and regular adaptation all over the arch, by inducing absorption in some places and deposit in others.

So much depends upon the general health and local condition of patients for whom artificial dentures are required that it is at least judicious not to promise too much. The dentist should rather moderate than excite the expectations of his patient as to the rapidity and facility of adapting and learning to use satisfactorily these substitutes for the natural organs. Not that we can overrate the advantages ultimately resulting from skillfully produced and intelligently used artificial dentures, but these qualities so seldom combine, that the percentum of really first-class work is woefully small, compared to the number of monstrosities unblushingly claiming to "rival the finest productions of nature's immaculate workmanship."

Let us first learn, my dear fellow-laborers, to appreciate these productions of nature, and my word for it, we will be able better to appreciate the attainments which enables any to even approximate such perfection.—*Cosmos.*

